# Biochemical and structural basis of sialic acid utilization by gut microbes

**DOI:** 10.1016/j.jbc.2023.102989

**Published:** 2023-02-08

**Authors:** Andrew Bell, Emmanuele Severi, C David Owen, Dimitrios Latousakis, Nathalie Juge

**Affiliations:** 1 Quadram Institute Bioscience, Gut Microbes and Health Institute Strategic Programme, Norwich, United Kingdom; 2 Microbes in Health and Disease, Biosciences Institute, Newcastle University, Newcastle upon Tyne, United Kingdom; 3 Diamond Light Source Ltd, Diamond House, Harwell Science and Innovation Campus, Didcot, United Kingdom

**Keywords:** sialic acid, sialidase, sialic acid transporters, sialic acid metabolism, sialic acid metabolic enzymes, gut microbiota, mucin glycosylation, mucus, enteric pathogens, CasD1, capsule structure 1 domain 1, CM, cytoplasmic membrane, GF, germ-free, GI, gastrointestinal, HMO, human milk oligosaccharide, ITC, isothermal titration calorimetry, LST, sialyllacto-N-tetraose, Neu5Ac, N-acetylneuraminic acid, Neu5Gc, N-glycolylneuraminic acid, OM, outer membrane, SBP, solute-binding protein, STD NMR, saturation transfer difference nuclear magnetic resonance spectroscopy, TM, transmembrane

## Abstract

The human gastrointestinal (GI) tract harbors diverse microbial communities collectively known as the gut microbiota that exert a profound impact on human health and disease. The repartition and availability of sialic acid derivatives in the gut have a significant impact on the modulation of gut microbes and host susceptibility to infection and inflammation. Although N-acetylneuraminic acid (Neu5Ac) is the main form of sialic acids in humans, the sialic acid family regroups more than 50 structurally and chemically distinct modified derivatives. In the GI tract, sialic acids are found in the terminal location of mucin glycan chains constituting the mucus layer and also come from human milk oligosaccharides in the infant gut or from meat-based foods in adults. The repartition of sialic acid in the GI tract influences the gut microbiota composition and pathogen colonization. In this review, we provide an update on the mechanisms underpinning sialic acid utilization by gut microbes, focusing on sialidases, transporters, and metabolic enzymes.

## Introduction: sialic acids in the gut

**Figure 1:**
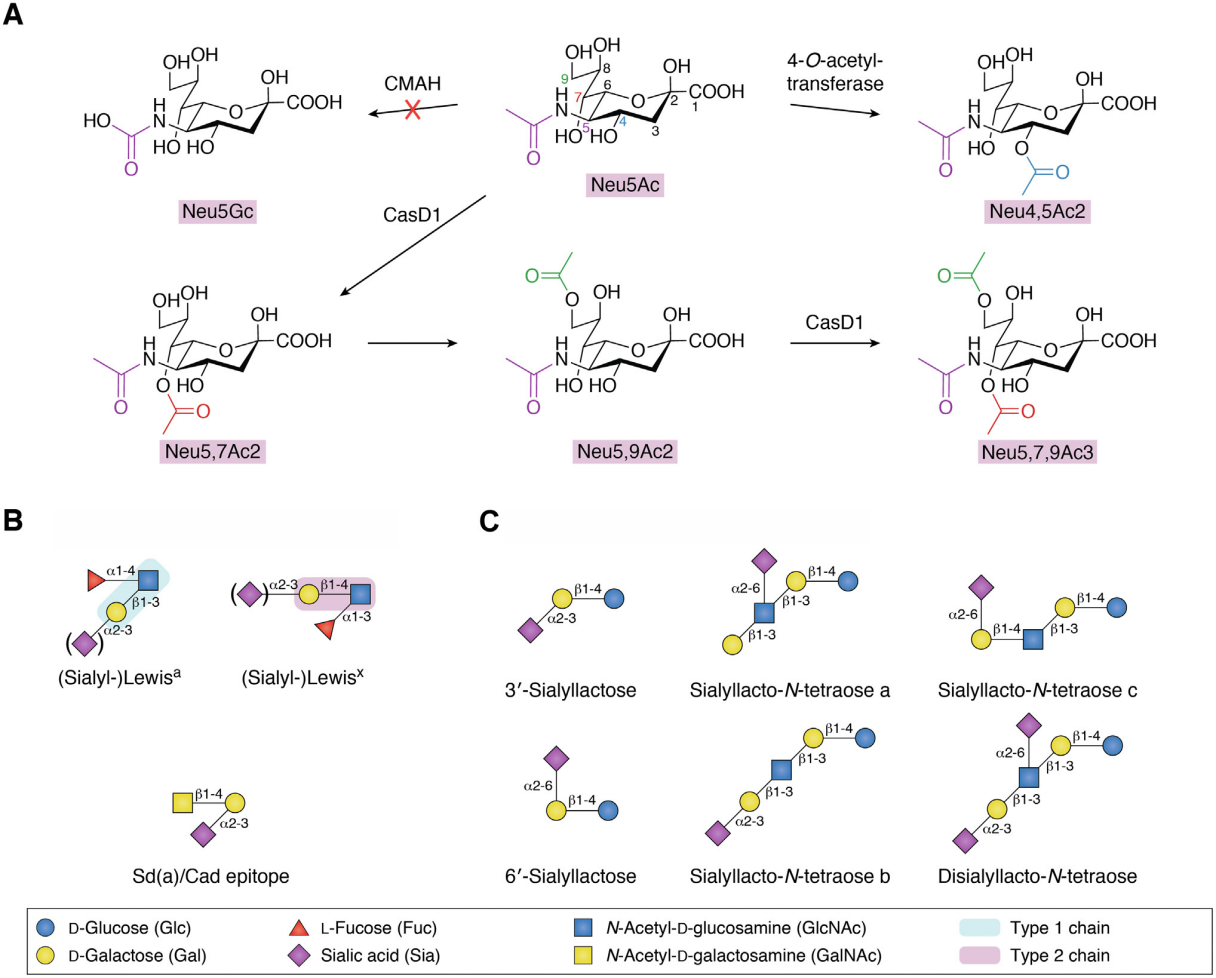
**Chemical structures of sialic acid derivatives found in the gut.***A*, common Neu5Ac modifications. The substrate of CasD1 and CMAH is CMP-Neu5Ac, but free forms of sialic acids have been drawn for simplicity. *B*, main sialylated mucin glycan epitopes and *C*, main sialylated human milk oligosaccharides. The monosaccharide depictions follow the standard Symbol Nomenclature for Glycans (145).

Sialic acids are a large family of nine-carbon sugars covering more than 50 structurally and chemically distinct modified forms. Sialic acids are abundant components of vertebrate glycoproteins, glycolipids, and milk oligosaccharides, as well as on some microbial surface glycans, mediating diverse functional roles including glycan–protein, cell–cell, and microbe–host recognition (1). The most common form of sialic acid in humans is N-acetylneuraminic acid (2-keto-5-acetamido-3,5-dideoxy-D-glycero-D-galactononulopyranos-1-onic acid) (Neu5Ac). Neu5Ac can be further modified by the addition of O-acetyl modifications at the C-4, -7, -8, and -9 positions or by the hydroxylation of the N-acetyl group at C-5 to form N-glycolylneuraminic acid (Neu5Gc). So far, capsule structure 1 domain 1 (CasD1) is the only enzyme identified in humans that can O-acetylate Neu5Ac, and its function has been directly implicated in the modification of positions C-7 and C-9 (2, 3). In addition, it has been shown that acetyl groups in position 7 can spontaneously migrate to positions C-8 and C-9, with the latter being the most stable form (4). It is thought that CasD1 adds acetyl groups at C-7, from which it migrates to the C-9 position (Neu5,9Ac2) under physiological conditions (4, 5). This would allow an additional acetyl group to be added by CasD1 to C-7 to form the di-O-acetylated Neu5,7,9Ac3 (2), although this is yet to be confirmed experimentally. CasD1 has been shown to act directly on the cytidine-5-monophospho (CMP)-Neu5Ac prior to the transfer of the sialic acid moiety onto the acceptor target, suggesting that Neu5Ac is modified prior to sialylation (3); however, the ability of CasD1 to accommodate other acceptors such as sialoglycans has not been investigated. Additional O-acetyl modifications can also be incorporated at the C-4 position to generate Neu4,5Ac2, but the enzyme catalyzing this reaction remains to be identified (Fig. 1*A*). O-Sulfation of sialic acid has been less studied as compared with O-acetylation, and although sulfated sialic acids have recently been reported in vertebrate cells and tissues by immunodetection, and the sialate O-sulfotransferases, responsible for the sulfation identified, their occurrence in the gut remains to be determined (6). Given the diversity of sialic acid modifications in nature, the Symbol Nomenclature For Glycans rules have been expanded to represent this natural diversity (7). Humans lack the CMP-N-acetylneuraminic acid hydroxylase (CMAH) enzyme, which synthesizes the N-glycolyl modification in Neu5Gc, but Neu5Gc can be metabolically incorporated into human tissues from dietary sources rich in Neu5Gc such as red meat (8). In the gastrointestinal (GI) tract, sialic acids are found in the terminal location of mucin glycan chains constituting the mucus layer and in certain human milk oligosaccharides (HMOs) while a rare component of the cell surface of some bacterial species, as described below.

### Sialic acids of the GI tract

In the gut, a major source of sialic acid comes from mucins, which are the main structural components of the mucus layers covering the GI tract. Mucin 2, MUC2 (humans) and Muc2 (mice), is the major secreted mucin of both the small and large intestines. In the colon, where most of the gut microbiota resides, mucus is organized in a bilayer, with the outer layer harboring gut microbes and the inner layer protecting the underlying epithelium from luminal contents and bacterial invasion. Mucins are highly glycosylated proteins decorated with a diverse and complex array of O-glycan structures containing N-acetylgalactosamine (GalNAc), galactose (Gal), and N-acetylglucosamine (GlcNAc) and usually terminated by fucose (Fuc), sialic acid residues (Neu5Ac), and sulfate. Different combinations of, and linkages between, these monosaccharides result in characteristic glycan epitopes, such as blood groups A, B, and H and sialyl-Lewis epitopes (9). These carbohydrate-based blood groups impact not only fundamental areas of human biology and medicine including susceptibility to infection but also transplantation and transfusion, as recently reviewed (10) while contributing to shaping the gut microbiota composition (11, 12). Lewis (Le) antigens are constituted from two types of backbone structures, the type 1 chains ([composed of Gal-β(1,3)-GlcNAc) giving rise to Lewis^a^ (Le^a^), sialyl-Le^a^, and Lewis^b^ (Le^b^), and type 2 chains (composed of Gal-β(1,4)-GlcNAc, also termed N-acetyllactosamine [LacNAc]) giving rise to Lewis^x^ (Le^x^), sialyl-Le^x^, and Lewis^y^ (Le^y^). Further addition of a β1,4 GlcNAc onto the Gal residue of the α2,3 sialyl-lactosamine epitope forms the Sd(a)/Cad epitope (Fig. 1*B*). In the GI tract, these glycan epitopes provide an attachment site and source of nutrient for commensal and pathogenic bacteria while protecting from extensive degradation of the mucus layer (13, 14, 15, 16, 17).

Neu5Ac is the most abundant sialic acid in both human adult and fetal GI tract, with the level of expression of Neu5,8Ac2 in fetuses being higher compared with the adult intestine (18). Mucin glycosylation varies along the GI tract with a decreasing gradient of Fuc and ABH blood group expression and an increasing gradient of sialic acid from the ileum to the colon in humans (19). For example, blood group H and A antigenic determinants were shown to be present exclusively in the ileum and cecum, whereas blood group Sd(a)/Cad-related epitopes were found to increase along the length of the colon (20). The biosynthetic pathway leading to the Sd(a) antigen includes the intermediate structure sialyl-N-acetyllactosamine (Neu5Acα-2,3Galβ1,3/4GlcNAcβ1-), and this represents an important branch point in the pathway as it may be converted to the Sd(a) antigen, sialyl-Lewis^a^, or sialyl-Lewis^x^ (21). In healthy adults, nano-liquid chromatography/mass spectrometry analysis of the MUC2 O-glycans of the sigmoid colon revealed the presence of more than 100 complex O-linked oligosaccharides. Most of the oligosaccharides were based on the core 3 structure (GlcNAcβ-1,3GalNAc), one of the four mucin core structures found in the intestine (22), with sialic acid at the 6-position of the GalNAc, and the substructure Galβ1,3/4-GlcNAcβ1,3(NeuAc-6)GalNAcol was found in most glycans. The most abundant components were -Gal-(Fuc)GlcNAc-3(NeuAc-6)GalNAcol, GalNAc-(NeuAc-)Gal-4/3GlcNAc-3(NeuAc-6)GalNAcol, GalNAc-3(NeuAc-6) GalNAcol, and GlcNAc-3(NeuAc-6)GalNAcol (23). In contrast to human adult intestinal mucins, no sialic acid or fucose gradient was observed from ileum to distal colon in the fetal intestine (18).

Owing to the invasiveness of procedures to access intestinal mucus in humans, a lot of the information regarding the composition and regulation of mucin glycosylation in the GI tract comes from investigations using mouse models. Many glycans in the mouse intestine were found to be mono- or disialylated with sialic acid linked by α2,3- and α2,6-glycosidic bonds (24). In contrast to the gradients in humans, sialylated structures dominate the murine small intestine while structures terminating with Fuc are increased in the colon (24). The Muc2 O-glycosylation patterns correlate with O-glycosyltransferase abundances in the epithelial cells along the intestine of mice (25). Six different sialyltransferases were detected, although their product specificity remains undefined (26). The main sialylation enzymes identified were St3gal6 and St6gal1, with the highest expression in the colon, and St3gal4, with the highest expression in the small intestine (25). Terminal sialylation of mucin glycans by ST6GALNAC1 (ST6) was recently found to be essential for mucus integrity and protecting against excessive bacterial proteolytic degradation (27). Analysis of sialic acid derivatives in the murine GI tract showed high levels of 9-O-acetyl, 7,9-O-acetyl, 4-O-acetyl, and Neu5Gc modifications. The acetylated forms Neu5,9Ac2, Neu5,7Ac2, and Neu5,7(8),9Ac3 have been reported in the epithelial cells, goblet cells, and mucus layers of stomach, small intestine, and colon. Colon showed the highest levels of total O-acetylation, representing ∼17% of sialic acid having one or more *O*-acetyl modifications, primarily 9-O-Ac, while the levels of 4-O-Ac sialic acid were generally low, making up ∼2% of sialic acid in the small intestine (duodenum). The high levels of 7,9-O*-* and 9-O-Ac found in the mouse colon were most likely associated with secreted mucus (28). Neu5Gc, Neu5Ac, and its modified acetylated forms, Neu5,7Ac2, Neu5,8Ac2, Neu5Gc9Ac, Neu5,9Ac2, and Neu5,7(8)9Ac3, were also detected in the cecum of germ-free (GF) and gnotobiotic mice (29). GF mice showed alteration in Muc2 glycan levels, and less sialylated glycans were found in GF mouse ileum and colon (25).

These structures may be influenced by the gut microbiota and pathogens by influencing synthesis/degradation of sialylated structures, although O-acetylation of sialic acid in the gut (Neu5,7Ac2 and Neu5,9Ac2) is partially resistant to the action of bacterial sialidases (30). Recent work identified 2,7-anhydro-Neu5Ac in the cecum of mice monocolonized with *Ruminococcus gnavus* ATCC 29149, a human gut symbiont with the capacity to release α2,3-linked sialic acid into 2,7-anhydro-Neu5Ac, through the action of an intramolecular *trans*-sialidase (IT-sialidase) (31, 32). Alterations in mucin glycosylation cause a disruption in gut homeostasis, contributing to a compromised intestinal barrier, increased susceptibility to infection, and colitis as demonstrated in mouse models (33, 34, 35). In patients with inflammatory bowel disease, it was further shown that the MUC2 glycosylation pattern was reversed to normal when a patient with active disease went into remission and that patients with strong alterations in the glycan pattern tended to have a more severe disease course (36). Glycoproteomic profiling and biochemical analysis of ST6 mutations identified in patients showed that decreased sialylation causes defective mucus and inflammatory bowel disease (27).

### Sialic acids from dietary source

Dietary sialic acid consumption (as monosaccharide or as part of certain HMOs or meat-based foods) benefits the growth of microorganisms with sialic acid metabolism capabilities, thereby influencing the gut microbiota composition (37).

HMOs are structurally similar to mucin oligosaccharides and represent a rich source of sialic acids for the gut microbiota of infants. HMOs have been classified into 13 core structures that consist of lactose, at the reducing end, elongated by β1,3-linked lacto-N-biose I (Galβ1,3GlcNAc, LNB, type 1 chain) and/or β1,3/6-linked N-acetyllactosamine (Galβ1,4GlcNAc, LacNAc, type 2 chain). These core structures are frequently modified by fucose and sialic acid residues *via* α1,2/3/4 and α2,3/6 linkages, respectively. Based on the presence of sialic acid, HMOs are divided into acidic oligosaccharides (which contain one or more sialic acid molecules) and neutral or fucosylated oligosaccharides (which do not contain sialic acid). The nature and concentration of sialylated HMOs in human milk varies between different ethnic groups and geographic study groups, and the course of lactation (for a review, see Hobbs *et al*., 2021 (38)). More than 55 structurally distinct sialylated HMOs have been characterized so far. The simplest sialylated oligosaccharides present in milk are trisaccharides, such as 3′-sialyllactose (3′-SL) and 6′-sialyllactose (6′-SL), which are formed by the addition of sialic acid *via* α2,3- and α2,6-glycosidic bonds to the galactose of lactose, respectively (Fig. 1*C*). The other oligosaccharides occurring in human milk containing α2,3- and/or α2,6-linked sialic acid are disialyllacto-N-tetraose and sialyllacto-N-tetraoses (LST) such as LSTa, LSTb, and LSTc (Fig. 1*C*). The majority of HMOs reach the colon undigested where they become substrates for the gut microbiota. Recent preclinical studies suggest that dietary supplementation with sialic acid or sialylated HMOs enhances brain development and performance (for recent reviews see (Hobbs *et al*., 2021 and Liu *et al*., 2022 (38, 39)). In addition, approximately 57% of N-glycans and most glycolipids in human milk are sialylated glycoconjugates that are involved in neurodevelopment and cognition (40). Moreover, the form of sialic acid is 100% Neu5Ac in human milk, which is about 25 to 80% higher than any commercially available infant formulas (41). Necrotizing enterocolitis, a fatal intestinal disorder in preterm infants, occurs 6 to 10 times more frequently in formula-fed infants compared with breast-fed infants, and both preclinical and clinical cohort studies pointed toward disialyllacto-N-tetraose as a biomarker or therapeutic target (42, 43).

Sialic acid in the adult gut can also come from dietary sources. A survey of sialic acid levels in foods quantified bound and free Neu5Gc and Neu5Ac in meat, dairy, seafood, vegetables, and fruits. According to this analysis, a great majority of the sialic acids were glycosidically bound and not free, even after cooking. Fruits and vegetables did not contain any sialic acids. Neu5Gc was found in moderate to high amounts in foods from mammals (cow, goat, sheep, pig, and bison) while no Neu5Gc was in poultry and eggs or in seafood (44). Overall, the highest levels of Neu5Gc among the red meats were in beef, which contains up to 231 μg of Neu5Gc per gram of meat, and the lowest amounts were seen in milk and milk products, with Neu5Gc levels ranging from 2 to 40 μg/g. Beef also contained the highest percent Neu5Gc of total sialic acid, and this Neu5Ac/Neu5Gc ratio may be relevant because of likely competition of Neu5Ac with Neu5Gc for incorporation into cells (44). Indeed, the bound form of Neu5Gc is bioavailable, undergoing metabolic incorporation into human tissues and the subsequent interaction with inflammation-provoking antibodies against this "xenoautoantigen" (for a review, see Alisson-Silva *et al*., 2016 (45)).

### Sialic acids on the microbial cell surface

Sialic acid in the gut is also found as part of the cell surface glycosylation of gut microbes, although most studies so far have focused on pathogenic bacteria responsible for gastroenteric infections or cancer such as strains of *Campylobacter jejuni* (46), *Escherichia coli* (47), *Helicobacter pylori* (48, 49), *Fusobacterium nucleatum* (50), *Hafnia alvei* (51), *Salmonella enterica* serovar Toucra (52) or *Streptococcus agalactiae* (53), as reviewed in Dudek *et al*., 2022 (54). In these strains, sialic acid can be found as part of lipooligosaccharides or O-chains of surface lipopolysaccharide, as well as O-linked on flagellar proteins (49, 55). Recently, the application of a sialic acid-based azide-containing probe N-acetyl-9-azido-9-deoxy-neuraminic acid (Neu5Ac9N3/Sia9N3) was used to selectively label sialic acid–presenting bacteria from a complex cultured human fecal microbiome. Using fluorescence activated cell sorting and 16S rRNA gene sequencing analyses, it was found that the Sia9N3-incorporating bacteria belonged to the *Escherichia* genus and a new strain of *E. coli* with Neu5Ac on its surface was identified (56). However, the mechanisms underpinning the capacity of gut commensal bacteria to self-decorate remain to be investigated; some may have the ability to scavenge host sialic acids while others may be able to synthesize them (with examples of both pathways seen in bacterial pathogens from other niches (57)). In addition, certain bacteria are able to switch their surface glycosylation profile in a phenomenon known as phase variation. For example, *C. jejuni* can express a variety of different sialyloligosaccharides in its core oligosaccharide, including mimics of gangliosides GM1, GD1a, GD3, and GT1a (58). The pattern of terminal glycosylation is determined by genetic variation in the lipooligosaccharides biosynthetic loci that include genes encoding CMP-NeuAc synthetase and a bifunctional sialyltransferase that can transfer sialic acid to galactose in α2,3 linkage as well as to sialic acid itself in α2,8 linkage (59, 60). The remodeling of cell-surface sialylated structures on microbes may also occur through microbial sialidase activity, although this is speculative at this stage. Today most studies on the role of cell-surface sialic acid in microbes focused on the role of Neu5Ac in bacterial pathogenesis by helping bacteria evading the host innate immunity response through molecular mimicry (54), but the role of microbial sialic acid as a potential metabolic substrate influencing the gut microbiota remains to be investigated.

## Influence of sialic acid on the interaction with gut microbes

Sialic acid in mucin glycan epitopes or HMOs provide nutritional and adhesion targets for gut microbes, as recently reviewed (38, 61). The capacity of gut microbes to consume sialic acid has been demonstrated using model organisms in mono- or cocultures and its implication on the composition of the gut microbiota community demonstrated using *in vitro* gut models or *in vivo* following sialic acid supplementation, as described below.

### Sialic acid as nutrient for gut microbes

The ability of individual gut bacteria species to utilize sialylated substrates has been tested in anaerobic cultures *in vitro* using 3′-SL and 6′-SL as the sole carbon source. Supplementation with 3′-SL and 6′-SL promoted moderate growth of *Bifidobacterium longum* JCM7007, 7009, 7010, 7011, 1272, 11347, ATCC15708; *Bacteroides vulgatus* ATCC8482; and *Bacteroides thetaiotaomicron* ATCC29148 while *Lactobacillus delbrueckii* ATCC7830 also consumed 6′-SL (62). In contrast, *R. gnavus* ATCC 29149 was able to utilize 3′-SL but not 6′-SL or lactose as the sole carbon source in *in vitro* anaerobic cultures (63). Depending on the species, the capacity of the strains to benefit from sialyllactose may result from their capacity to utilize lactose rather than sialic acid. This is particularly true for Bifidobacteria species such as *Bifidobacterium breve*, *Bifidobacterium infantis*, and *Bifidobacterium bifidum* (64). The released sialic acid could then serve as substrate for community members through cross-feeding, rather than the microbes releasing it. For example, *B. bifidum*–released sialic acid from mucins or HMOs can be utilized by *B. breve* to promote its growth (65). In addition all *B. breve* isolates from breast-fed infant feces could utilize sialylated HMOs to a certain extent, especially sialyllacto-N-tetraose (S-LNT) (66), as also reported for *B. longum* subsp. infantis ATCC15697 (67). All *B. breve* strains tested in this study showed a preferential consumption of acidic HMOs such as LSTb and monosialyllacto-N-hexaose, but not smaller HMOs, which may explain why growth on 3′-SL and 6′-SL was negligible (66). *B. thetaiotaomicron* strains can cleave Neu5Ac from sialylated HMOs, presumably to access underlying sugars, but are unable to catabolize it (68). This is also the case for *Akkermansia muciniphila* Muc^T^ (ATCC BAA-835) in the infant gut, which was shown to benefit from 3′-SL supplementation *in vitro*, but the liberated Neu5Ac from 3′-SL was not further catabolized by the bacterium (69). In addition, a recent study showed that there was a wide range of 6′-SL utilization across *A. muciniphila* strains, but in all strains, sialic acid accumulated in the culture medium and was not consumed when liberated from 6′-SL (70). The sialic acid released from the nonreducing end of the sugars enables access to the underlying sugars while also promoting the growth of sialic acid–metabolizing commensal species such as *Bacteroides fragilis* (68).

**Figure 2:**
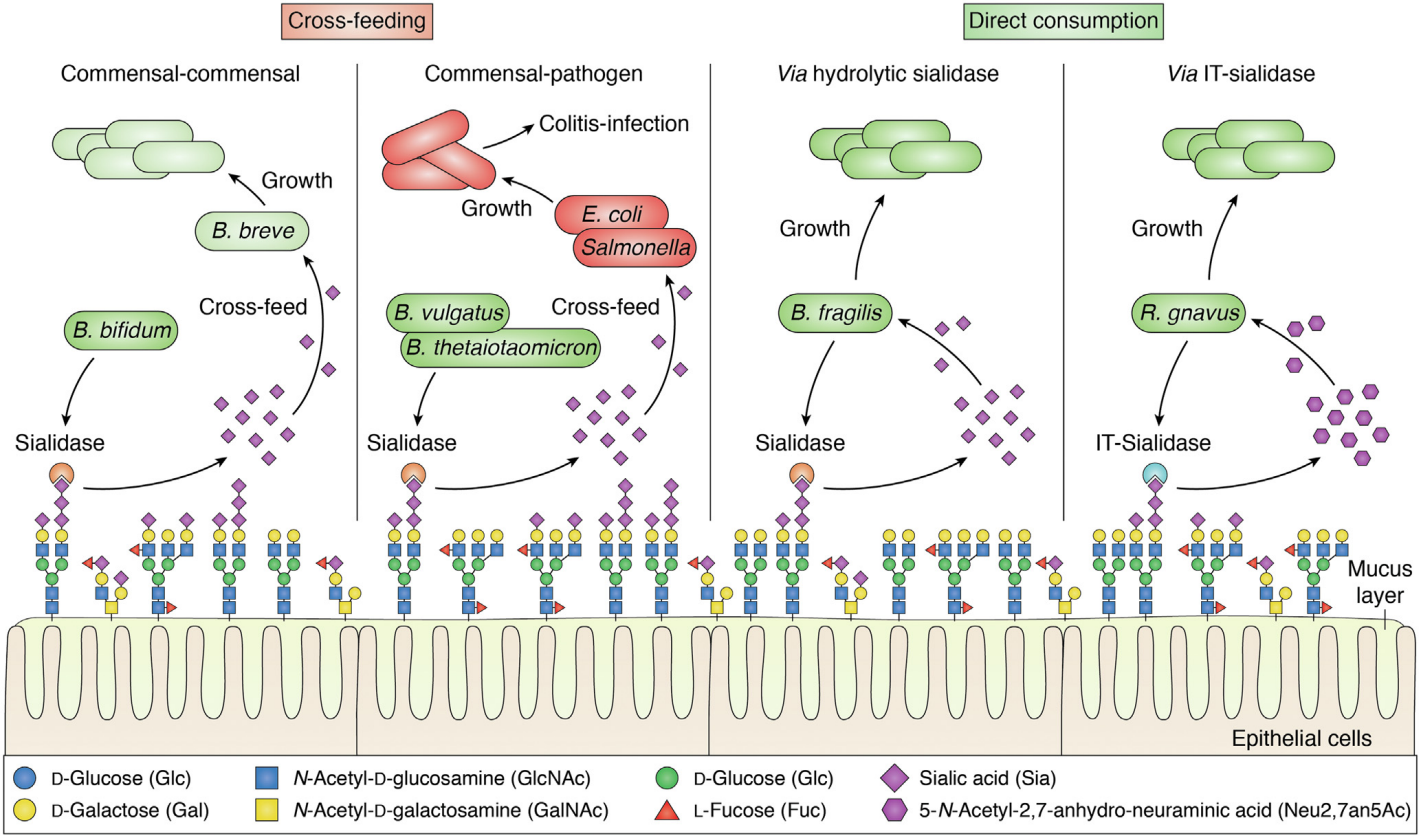
**Schematic representation of the role of mucin-derived sialic acid on gut homeostasis.** Sialic acid capping mucin glycan chains at the mucosal surfaces can be released by bacteria as Neu5Ac *via* hydrolytic sialidases or as 2,7-anhydro-Neu5Ac by IT-sialidases. The released sialic acids can then be scavenged by other microorganisms inhabiting the same niche through cross-feeding or consumed by the sialidase-producing bacteria depending on their sialic acid metabolism makeup. Examples of cross-feeding can occur between commensals or between commensals and pathogens.

From these studies, it is clear that the capacity of commensal microbes to utilize sialic acid as metabolic substrates provides them with a nutritional advantage to bacteria that have adapted to the GI mucosal environment. In addition, several pathogenic species of the Enterobacteriaceae family, such as *E. coli* and *S. enterica*, also thrive in a sialic acid–rich gut environment as demonstrated in mouse models. For example, antibiotic treatment led to a transient spike in liberated sialic acids, which promoted outgrowth of *Clostridioides difficile* and *Salmonella* pathogens, which do not encode sialidases but can metabolize free sialic acid (71). In mouse model of colitis, increased levels of sialidase activity from *B. vulgatus* led to outgrowth of *E. coli* in the gut, which was supported by data showing that, in contrast to free Neu5Ac, 3′-SL and 6′-SL did not support *E. coli* growth *in vitro* (72). Hence, sialic acids released in the gut by mucus-adapted bacteria such *A. muciniphila* or *B. thetaiotaomicron* can be scavenged by sialic acid–metabolizing commensals and potential pathogens, affecting gut homeostasis (Fig. 2).

### Sialic acid as modulator of the gut microbiota

*In vitro* fermentation and *in vivo* studies demonstrated that supplementation with sialic acid (Neu5Ac or Neu5Gc) or sialylated glycans can alter the overall gut microbiota composition. Supplementation of sialylated bovine milk oligosaccharides in preclinical models (gnotobiotic mice and piglets) showed a causal, microbiota-dependent relationship between sialylated bovine milk oligosaccharides and growth promotion (73). *In vitro* batch fermentation studies using fresh piglet cecal contents supplemented with [^13^C]-Neu5Ac caused significant microbial community changes with a relative increase in *Prevotella* and *Lactobacillus* species, accompanied by a reduction in the genera *Escherichia/Shigella, Ruminococcus,* and *Eubacterium*. Further inspection of isotopically labeled RNA sequences suggested that the labeled Neu5Ac was consumed by a wide range of bacteria with the genus *Prevotella* identified as the most prolific users (74). Significant microbiome differences were also observed in the proximal and distal colon of piglets fed with 6′-SL. Differences were attributed to an increase in bacterial taxa belonging to species *Collinsella aerofaciens* (phylum Actinobacteria), genera *Ruminococcus* and *Faecalibacterium* (phylum Firmicutes), and genus *Prevotella* (phylum Bacteroidetes) compared with piglets fed the control diet. Taxa belonging to families Enterobacteriaceae and Enterococcaceae (phylum Proteobacteria) and taxa belonging to family Lachnospiraceae and order Lactobacillales (phylum Firmicutes) were 2.3- and 4-fold lower, respectively, in 6′-SL-fed piglets than in controls (75). Supplementation with 6′-SL also increased the ganglioside-bound sialic acid in the brains of the piglets, thus providing essential nutrients for brain growth and neurodevelopment (76).

A recent study investigating the effects of Neu5Ac on gut morphology, liver function, and gut microbes mice showed dose-dependent changes in the gut microbiota composition as determined by 16S rDNA gene sequencing (77). At the phylum level, Firmicutes, Actinobacteriota, Gemmatimonadetes, and Chloroflexi were markedly enhanced. At the species level, *Staphylococcus lentus*, *Corynebacterium stationis*, *Jeotgalibaca* sp PTS2502, *Ignatzschineria indica*, *Sporosarcina pasteurii*, *Corynebacterium urealyticum*, *Facklamia tabacinasalis*, *Sporosarcina* sp HW10C2, and *Oblitimonas alkaliphila* were notably increased in the group receiving the highest dose of sialic acid (4.8 mmol ml^−1^) while *Erysipelatoclostridium ramosum*, *Blautia* sp YL58, *B. thetaiotaomicron*, *Morganella morganii*, and *C. difficile* were enhanced in the group given a lower dose (1.6 mmol ml^−1^) (77), stressing the importance of sialic acid availability in shaping the gut microbiota.

A Neu5Gc-rich diet also induces changes in the gut microbiota, with Bacteroidales and Clostridiales responding the most. Genome assembling of mouse and human shotgun metagenomic sequencing identified bacterial sialidases with previously unobserved substrate preference for Neu5Gc-containing glycans (78). The authors proposed that the release of Neu5Gc from red meat by bacterial sialidases could reduce the risk of inflammatory diseases associated with red meat consumption, including colorectal cancer and atherosclerosis (78). In addition, sialic acid as a nutrient source for bacteria, such as *Bacteroides* and *Clostridium*, and sialidase-released Neu5Gc from red meat reduced the risk of inflammatory diseases *via* the gut microbiota (78). A recent bioinformatics study analyzed the distribution of sialic acid utilization pathways and sialidase genes across a reference set of 2662 genomes representing ∼700 species and ∼200 genera of bacteria from the human gut (37). Approximately 1040 strains were predicted as Neu5Ac-utilizing strains, representing ∼80 bacterial genera. Among these, a sialidase was identified in 40% of the strains, including prominent colonic bacteria from the *Akkermansia*, *Bacteroides*, *Bifidobacterium*, *Clostridium*, *Flavonifractor*, *Parabacteroides*, and *Prevotella* genera. Another subgroup of strains that lack a sialidase but are capable of sialic acid utilization includes human gut symbionts such as *Anaerococcus*, *Blautia*, *Escherichia*, *Eubacterium*, *Faecalibacterium*, and *Fusobacterium* and also a number of opportunistic pathogens including *Clostridioides*, *Staphylococcus*, and *Streptococcus* spp. Finally, ∼100 strains from 27 microbial genera were shown to possess a sialidase but apparently lack the sialic acid utilization capability. These included 18 *Bacteroides* strains (*e.g.*, *B. faecis*, *B. intestinalis*, *B. thetaiotaomicron*), 6 *Porphyromonas* strains, and 6 *Coprobacillus* strains (37). These data underscore the importance of sialic acid in establishing metabolic networks within the gut microbial communities.

Collectively these studies indicate that the gut microbiota can be significantly changed in response to supplementation with dietary sialic acid or sialylated glycans and point toward the importance of sialic acid catabolism and metabolism by gut bacteria for gut homeostasis and brain development.

## Structural basis for sialic acid metabolism by gut microbes

The capacity of gut bacteria to consume sialic acids relies on their ability to liberate sialic acids from glycoproteins or oligosaccharides and transport them into the cells, where they are further metabolized. In bacteria, the genes involved in the metabolism of sialic acid are usually found together in so-called *nan* clusters. The following section describes the biochemical and structural properties of the main sialic acid enzymes and transporters involved in this pathway.

### Sialidases

The first step in sialic acid catabolism by gut microbes is carried out by sialidases that cleave these terminal residues from sialoconjugate substrates. Gut microbial sialidases belong to the glycoside hydrolase GH33 family of the CAZy database (www.cazy.org) (79). Gut bacteria that express sialidases include multiple species of *Clostridia* and *Bacteroides*, as well as specific strains of *Bifidobacterium, R. gnavus*, and *A. muciniphila* (80). Sialidases are generally functionally categorized into hydrolytic sialidases, *trans*-sialidases and intramolecular *trans*-sialidases (IT-sialidases), although no *trans*-sialidases have been reported in gut bacteria (see Table 1).

**Table 1:** List of functionally characterized sialic acid enzymes and transporters from gut bacteria

Protein class	Name	Bacteria^a^	Uniprot/GenBank	PDB	References
Sialidase					
	Hydrolytic sialidase (*Bt*NanH)	*B. thetaiotaomicron* VPI-5482	Q8AAK9	4BBW	(61)
	*Am*NanH	*A. muciniphilla* ATCC BAA-835	B2UPI3, B2UPI5, B2ULI1, B2UN42		(48, 62)
	*Sm*NanH	*S. multivorum*			(63)
	*Sh*NanH	*Sphingobacterium* sp. HMA12	WP_104384225.1		(64)
					
	Intramolecular *trans*-sialidase (*Rg*NanH)	*R. gnavus* ATCC 29149	A0A829NK98	4X47	(15, 16)
				4X49	
				4X4A	
				4X6K	
**O-acetylesterase/sialidase fusion**					
	*Bb*SiaBb1	*B. bifidum* JCM1254	AB278566.1		(72)
					
**Sialate O-acetylesterase**					(67)
	*Ec*NanS	*E. coli* 0175-H7	A0A0H3JKI3	3PT5	
	*Bf*EstA	*B. fragilis* NCTC 9343	A0A380YZM5		(69)
	*Pv*SAE	*P. vulgatus* ATCC 8482			(71)
**Mutarotase**					
	*Ec*NanM	*E. coli* BW25113	P39371	2UVK	(73)
**Transporters**					
Cytoplasmic membrane (CM)					
	**TRAP (ST2)**				
	apo-*Vc*SiaP	*V. cholerae*	Q9KR64	4MAG	(86)
	*Pm*SiaP+Neu5Ac	*P. multocida*	A0A2K0XYW6	4MMP	(86)
	apo-*Vc*SiaP (spin-labeled)	*V. cholerae* O1 El Tor		5LTC	(83)
	*Vc*SiaP+Neu5Ac	*V. cholerae* O1 El Tor		7A5Q	(85)
	*Vc*SiaP[R125A]+synthetic peptide (spin labeled)	*V. cholerae* O1 El Tor		7A5C	(85)
	**ABC (ST7)**				
	*Rg*SBP(SAT2)	*R. gnavus* ATCC 29149	WP_004843638.1		(15)
	**MFS (ST1)**				
	*Ec*NanT	*E. coli* BW25113	P41036		(84)
Outer membrane (OM)					
	*Ec*NanC	*E. coli* K12	P69856	2WJQ	(100)
	*Ec*NanC	*E. coli* K12		2WJR	(100)
	*Bf*NanU	*B. fragilis* NCTC 9343	Q5LEN2	4L7T	(102)
					
**Aldolases**					
	*Pm*NanA	*P. multocida*	Q9CKB0	4IMD	(112)
				4IMG	
				4IMC	
				4IME	
				4IMF	
	*Cp*NanA	*C. perfringens* str 13	Q9S4K9		(111, 113)
	*Lp*NanA	*L. plantarum* ATCC BAA-793	P59407		(110)
	*Ec*NanA	*E. coli* K12	P0A6L4	1NAL	(114)
				4BWL	
				2WNN	
				2WNQ	
				2WPB	
		*R. gnavus* ATCC29149	A7B555	6RAB	(15)
				6RD1	
				6RB7	
		*Sphingobacterium*			(115)
	*Bf*NanL	*B. fragilis* NCTC 9343	Q5LEN8		(104)
					
**Anomerase**					
	*Ec*NanQ (YhcH)	*E. coli* K12	P45424		(99)
**Oxidoreductase**					
	*Rg*NanOx	*R. gnavus* ATCC 29149	A0A2N5NNS3	6Z3B	(16)
				6Z3C	
	*Ec*NanY (YjhC)	*E. coli* K12	P39353	6O15	(78)
**Kinase**					
	*Fn*NanK (ManNAc-6P kinase)	*F. nucleatum*	Q8RDN7	5NCK	(116)
	*Bf*RokA (GlcNAc kinase)	*B. fragilis* NCTC 9343	Q5GBH5		(118)
**Epimerase**					
	*Cp*NanE (ManNAc-6P epimerase)	*C. perfringens* str 13	Q8XNZ3	4UTW	(117)
	*Bf*NanE-II (AGE epimerase)	*B. fragilis* NCTC9343	WP_010992631		(104)

PDB, Protein Data Bank.

a Strain name is provided when specified in the reference.

#### Hydrolytic sialidases

**Figure 3:**
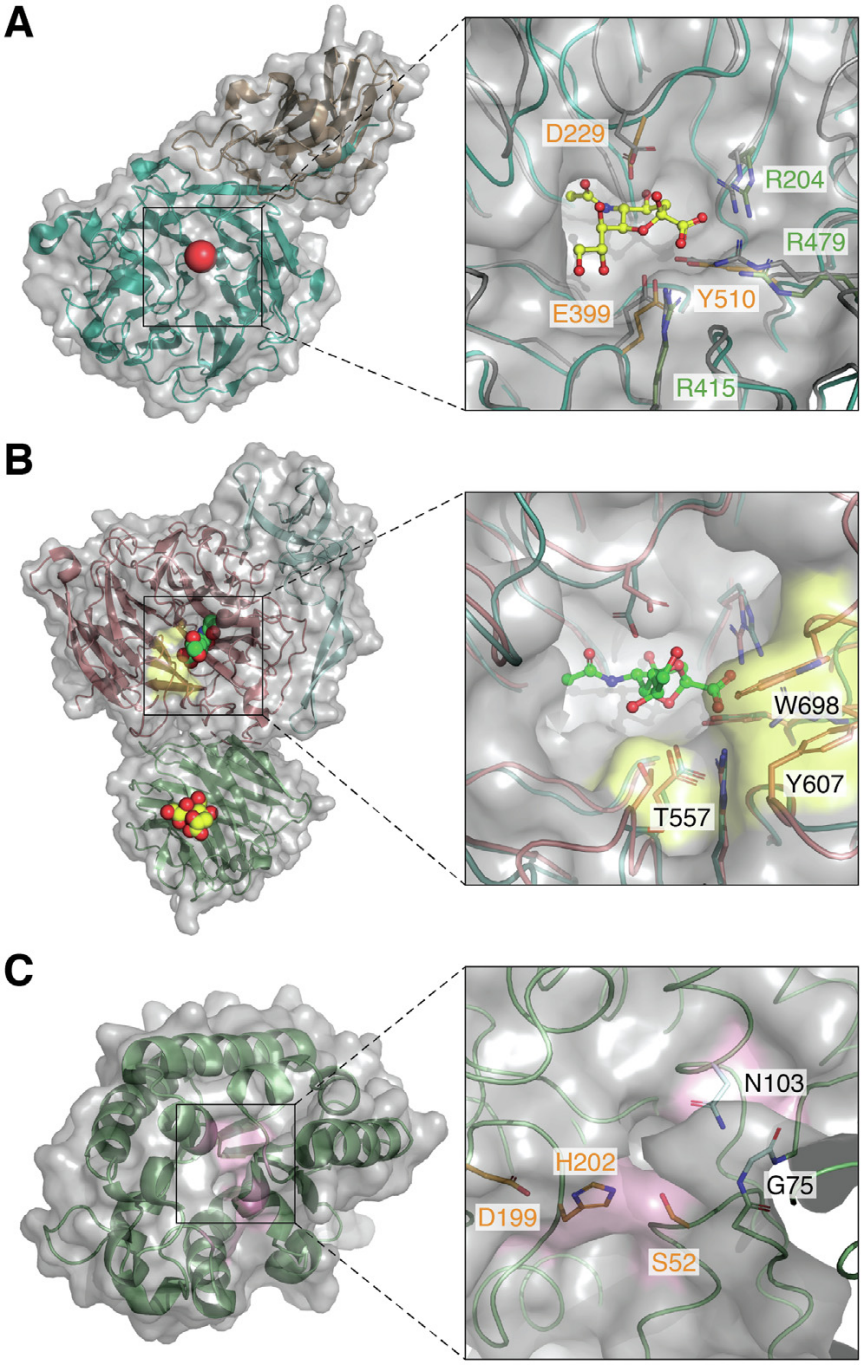
**Structural characteristics of sialic acid catabolic enzymes from gut bacteria.***A*, crystal structure of *B. thetaiotaomicron* VPI-5482 sialidase with catalytic domain in teal and proposed CBM40 domain in wheat (PDB 4BBW). The active site is indicated by a *red sphere.* Close-up of the active site shows the catalytic residues in *orange* and the arginine triad in *green.* In gray is the aligned crystal structure of *S. pneumoniae* NanA (PDB 2YA5) with sialic acid bound in *yellow.B*, composite structure of *R. gnavus* IT-sialidase *Rg*NanH. The catalytic domain in complex with 2,7-anhydro-Neu5Ac and inserted domain, *pink and light blue*, respectively (PDB 4X4A), and in *green* the CBM40 domain in complex with 2,3-sialyllactose (PDB 6ER3). The residues responsible for reaction specificity are highlighted with a *yellow* surface. The VPI-5482 sialidase is aligned in teal for comparison. *C*, 9-O-acetylesterase from *P. vulgatus* (PDB: 7PZG). The SGNH motif is highlighted with a *pink* surface and the canonical catalytic in *orange* in the active-site close-up. PDB, Protein Data Bank.

Hydrolytic sialidases are most common among gut microbes and have a broad substrate specificity for α2,3, α2,6- and α2,8-linked sialic acids. The gut microbial GH33 sialidases characterized to date use a retaining acid/base-catalyzed double displacement mechanism whereby a sialyl-enzyme intermediate is formed with an active site Tyr nucleophile; in hydrolytic sialidases a water molecule enters to facilitate the deglycosylation step, yielding the cleaved sialic acid with net retention of configuration (80). *Bacteroides* species are often found to encode hydrolytic sialidases but often lack the *nan* operon required for sialic acid utilization (37) (see section Sialic acid metabolic enzymes). The *B. thetaiotaomicron* VPI-5482 sialidase has been shown to have a broad substrate specificity toward α2,3, 2,6- and 2,8-linked sialic acid. The crystal structure of the enzyme shows a wide binding groove accommodating a range of substrates with Tyr510 and Glu399 as the catalytic pair (81) (Fig. 3*A*). Since *B. thetaiotaomicron* VPI-5482 does not metabolize sialic acid, the release of sialic acid is presumed to give greater access to the underlying glycans, which *B. thetaiotaomicron* can then access as a source of nutrients and metabolize further (81) (see section Sialic acid as nutrient for gut microbes).

*A. muciniphila* encodes sialidases that are capable of cleaving α2,3- or α2,6-linked sialic acid, and two were identified in proteomic datasets for *A. muciniphila* DSM 22959 grown in the presence of mucin glycans (69). Four sialidases from the same strain were biochemically characterized, each able to cleave α2,3- or 2,6-linked Neu5Ac, Neu5Gc, and N-propanoyl-neuraminic acid, and reduced activity on Kdn (2-Keto-3-deoxy-D-glycero-D-galacto-nononic acid, “deaminated sialic acid”) for three of the four enzymes (82). Like *B. thetaiotaomicron*, *A. muciniphila* ATCC-BAA-835 lacks the metabolic enzymes to break down sialic acid, so the sialidase activity is proposed to grant access to underlying structures, with sialic acid being free to potentially benefit other microbes, making these organisms key in establishing bacterial communities in the mucosal niche (69).

While most sialidase research focuses on Neu5Ac, some recent studies have examined the activity of gut microbe sialidases on other sialic acid derivatives. Genome assembling of mouse and human shotgun metagenomic sequencing identified Neu5Gc-preferential sialidases, with four of the five selected *Bacteroides* sialidases displaying preferential release of Neu5Gc over Neu5Ac (78). Sialidases from *Sphingobacterium* have been shown to release Kdn, with a characterized KDNase from *Sphingobacterium multivorum* shown to catalyze the release of Kdn but not Neu5Ac (83). More recently the gram-negative *Sphingobacterium* sp. HMA12 sialidase was characterized and showed a broad substrate specificity toward sialylated glycans, being active on α2,3/6/8/9 linkages as well as cleaving Kdn, Neu5Ac, and Neu5Gc moieties (84).

Although sialic acid can be modified by sulfates (6), the carbohydrate sulfatases from the gut microbiota characterized to date on colonic mucin O-glycans were found to cleave specifically sulfation of 3S-Gal or 6S-GlcNAc, and to our knowledge there is no report on microbial sulfatases active on sulfated sialic acid (85). Further exploration of sialidase specificity toward other sialic acid modifications found in the gut will undoubtedly reveal more novel microbial strategies to harvest sialic acids and colonize the gut.

#### IT-sialidases

As well as hydrolytic sialidases, one example of an IT-sialidase has been characterized in the gut symbiont *R. gnavus* ATCC 29149 (31, 32). The unique feature of IT-sialidases is that the O7-hydroxy group of the bound sialic acid glycerol group attacks the C2 atom leading to the release of 2,7-anhydro-Neu5Ac, as opposed to water in the case of hydrolytic sialidases (86, 87). This is likely due to the exclusion of water from the active site of the enzyme due to the architecture of the binding site (80). The crystal structure of *R. gnavus* IT-sialidase, *Rg*NanH, has been solved in complex with 2,7-anhydro-Neu5Ac (32). Its catalytic domain adopts the canonical six-bladed β-propeller fold of GH33; the active site contains the Arg triad (R257, R637, R575) as well as functional Glu559 and Tyr677 residues (Fig. 3*B*). In contrast to hydrolytic sialidases, a hydrophobic stack formed by Tyr607, Trp698, and Thr557 is likely responsible for the α2,3 specificity of IT-sialidases as well as creating a hydrophobic region, promoting nucleophile attack by the glycerol group opposed to water (32). *R. gnavus* IT-sialidase harbors a carbohydrate-binding module belonging to the CBM40 family (www.cazy.org). *Rg*NanH_CBM40 (*RgC*BM40) displays the canonical CBM40 β-sandwich fold as determined by X-ray crystallography and broad specificity toward sialoglycans with millimolar binding affinity toward α2,3- or α2,6-sialyllactose as shown by glycan arrays, saturation transfer difference nuclear magnetic resonance spectroscopy (STD NMR), and isothermal titration calorimetry (ITC) (88). Together, these biochemical and structural insights support the role of IT-sialidase in contributing to the location of *R. gnavus* ATCC29149 in sialic acid–rich region of the gut such as intestinal mucus, as further demonstrated in mouse models (31).

### Sialate O-acetylesterases

Neu5Ac modified with an O-acetyl group is generally resistant to release by sialidases. However, recent studies have shown that gut bacteria can produce O-acetylesterases to remove acetyl groups (Table 1). The O-acetylesterase from *E. coli* O175-H7, NanS, has been structurally characterized, showing a monomeric protein adopting a canonical α/β-hydrolase SGNH fold (89). *In vitro* NanS is active against Neu5,9Ac_2_ in free and conjugated forms (89). Site-directed mutagenesis confirmed the role in catalysis for the active site Ser and His residues characteristic of the SGNH fold, but the acidic residue needed to complete the canonical catalytic triad of SGNH hydrolases remains to be identified (89). NanS function is essential for *E. coli* growth on Neu5,9Ac_2_, providing indirect evidence that the *E. coli* sialic acid transporter NanT (discussed below) cannot recognize this form of sialic acid as a substrate (90).

*O*-acetylesterases have also been identified in *Bacteroides* species in the gut. For example, *B. fragilis* NCTC 9343 O-acetylesterase EstA has been shown to remove 9-O-acetyl esterifications, allowing sialidases to release Neu5Ac, which in turns promotes *in vitro* growth of *E. coli* (91). This could provide another example by which bacterial interactions share metabolic capabilities *in vivo* (91). This work also revealed that 7-O-acetylation is resistant to the action of the EstA O-acetylesterase and may therefore contribute to the integrity of mucin glycan chains. However, upon spontaneous migration to the 9-carbon position (9-O-acetylated sialic acid) (92), the O-acetyl ester becomes susceptible to the action of EstA, which may lead to changes in mucin glycan accessibility by the gut microbiota (69). Recently, the crystal structure of the 9-O-acetylesterase was determined from the gut symbiont *Phocaeicola vulgatus* (formerly *Bacteroides vulgatus*), revealing a canonical Ser-His-Asp catalytic triad, and flexibility in an N-terminal α-helix enabling the active site to accommodate large oligosaccharide substrates (93) (Fig. 3*C*). The binding pocket also suggests that the enzyme can accommodate Neu5Gc and acetylation at the 7-position, although this is yet to be characterized experimentally. An O-acetylesterase/sialidase fusion enzyme has also been reported in *B. bifidum*, the dual activity allowing removal of 9-O-acetyl group before the GH33 sialidase activity can remove Neu5Ac (94).

### Mutarotase NanM

Neu5Ac newly released by retaining GH33 sialidases is present predominantly as the α-anomer, which then only slowly mutarotates into the β-anomer accounting for ca 90% total Neu5Ac at equilibrium. *E. coli* K12 BW25113 has been shown to produce a mutarotase (epimerase), NanM (Table 1), which accelerates this process by increasing the equilibration rate between the α- and β-anomers of Neu5Ac, as shown by NMR on purified NanM, so that newly produced α-Neu5Ac released by sialidase action is promptly converted into the β-anomer (95). NanM is a soluble homodimeric protein formed by two six-bladed β-propellers, as determined by X-ray crystallography and analytical ultracentrifugation (95). Site-directed mutagenesis allowed the identification of a putative catalytic site located at one end of the propeller’s central cavity within each protomer (95). [^14^C]-Neu5Ac uptake assays and growth experiments provided evidence that NanM, located in the periplasm thus acting upstream of the transporter, might increase the efficiency of Neu5Ac uptake by the sialic acid transporter NanT (95). At the genetic level, the Neu5Ac mutarotase NanM is part of the Neu5Ac-inducible operon *nanCMS*, which also codes for the Neu5,9Ac2 esterase NanS (above) and the sialoporin NanC (which is discussed below). While the above studies are limited to *E. coli*, the widespread distribution of NanM homologues and various sialate O-acetylesterases among bacteria (95, 96, 97) underscores the need for sialic acid to be processed before being entering the cells.

### Sialic acid transporters

Once sialic acid is released, many gram-negative and gram-positive bacteria can acquire it using specific transport systems found both in the cytoplasmic membrane (CM) and, when present, in the outer membrane (OM) (98) (Table 1).

#### CM sialic acid transporters

**Figure 4:**
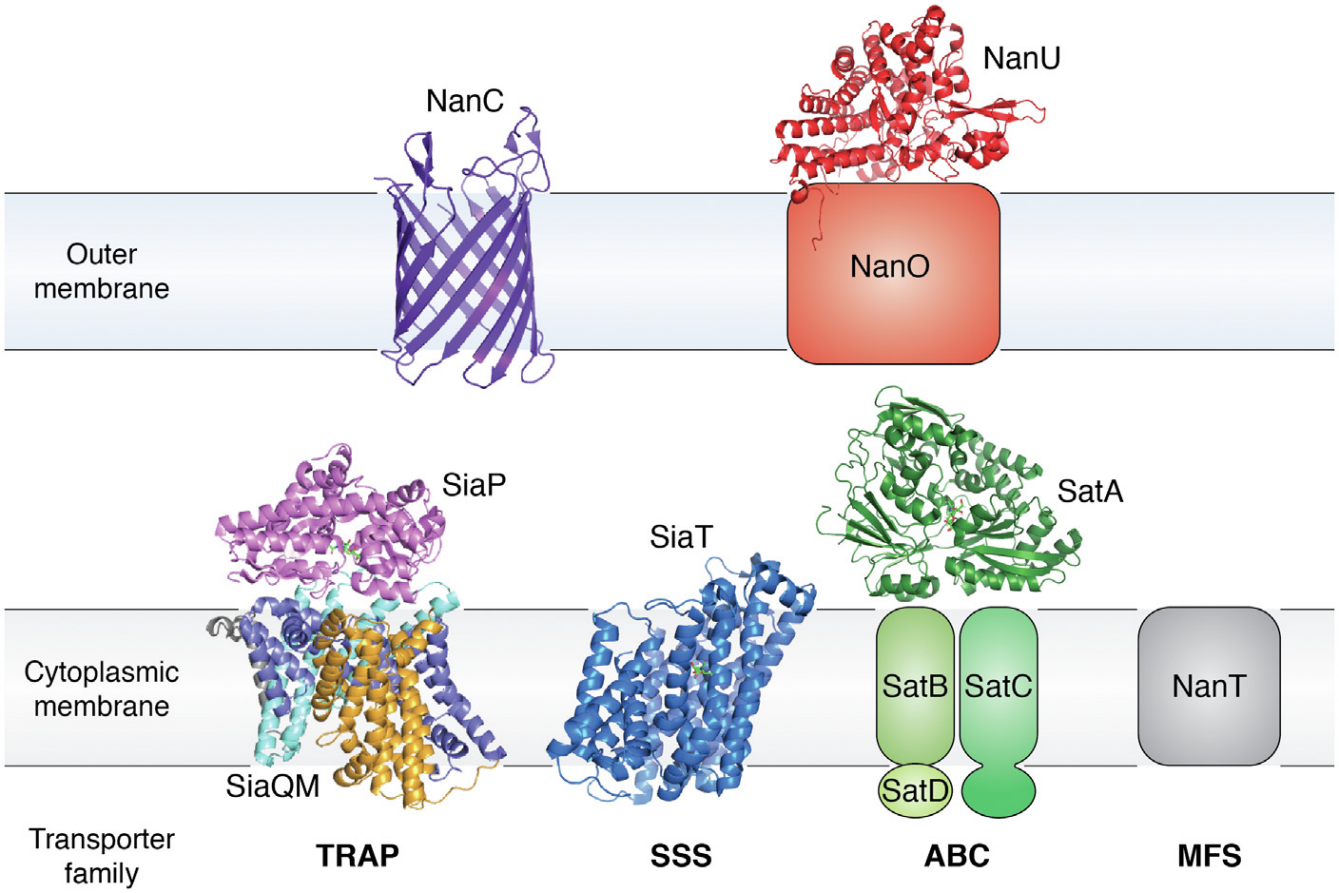
**Crystal structures of bacterial sialic acid transporters.** Bacteria use different transporters for sialic acid transport through the cytoplasmic membrane and, when present, the outer membrane. Most available structures concern transporters from organisms inhabiting niches other than the gut (see text), but crystal structures from gut microbes (namely, *B. fragilis*, *E. coli*, *V. cholerae*) have also been reported (see Table 1). Cytoplasmic membrane sialic acid transporters belong to either one of four superfamilies of transporters: TRAP, SSS, ABC, and MFS. Solved structures of complete transporters are from the TRAP transporter SiaPQM from *H. influenzae* (PDB 3B50, 7QE5) and the SSS transporter SiaT from *P. mirabilis* (PDB 5NVA). The only example of an ABC transporter crystal structure is SatA, the solute-binding protein of SatABCD from *H. ducreyi* (PDB 5Z99). To date no structure has been solved for any of the three different families of MFS transporters (NanT/X, NanZ, NanG). Outer membrane systems with solved structures include the sialoporin NanC from *E. coli* K12 (PDB 2WJR) and the SusD-family protein NanU (PDB 47LT), which is part of the SusCD-like system NanOU of *B. fragilis* NCTC 9343. In the model of the tripartite SiaPQM, the elevator domain of SiaM is depicted in yellow, while the stator domain formed at the Q-M interface is in cyan (SiaQ) and indigo (SiaM). See main text for references and structural and mechanistic details on all transporters. Figure adapted from Thomas, 2016 (98). PDB, Protein Data Bank.

Commensal and pathogenic bacteria inhabiting mucosal surfaces use a variety of CM transporters for the acquisition of sialic acid (97, 98, 99). These belong to one of four superfamilies of prokaryotic transporters, namely, TRAP (tripartite ATP-independent periplasmic), SSS (sodium-solute symporters), ABC (ATP-binding cassette), and MFS (major facilitator superfamily) transporters (Fig. 4). While ABC transporters are primary systems that use ATP hydrolysis for function, all others are secondary systems using ion gradients. CM sialic acid transporters are further classified based on phylogeny into eight independently evolved families (named ST1-8) differing by structural–functional features not accounted for at the superfamily level (97). CM transporters characterized so far have been mostly studied for their capacity to transport Neu5Ac (with some able to take up Neu5G and/or Kdn too) (97) or anhydro-Neu5Ac (31, 100). The most extensively studied sialic acid transporters to date are the TRAP transporter SiaPQM and the SSS transporter SiaT, which are the only two reports of a complete structure solved including the transmembrane (TM) domain(s) (101, 102) (Table 1).

##### TRAP transporters

SiaPQM (ST2) is a multicomponent system made of three proteins, all essential for function: the extracytoplasmic solute-binding protein (SBP), SiaP, performing the initial high-affinity capture of Neu5Ac before delivery to the membrane components, and the two TM proteins SiaQ and SiaM together forming a sodium-dependent secondary transporter of the “elevator” type, *i.e.*, a transporter with a mobile domain that slides across the membrane as a rigid body and ferries the substrate along (103). In gut bacteria, SiaPQM systems from the pathogen *Vibrio cholerae* O1 El Tor and related Vibrionaceae have been extensively studied (104, 105, 106, 107), but orthologues have also been characterized from other host-associated microorganisms and from pathogens outside the gut including the respiratory pathogen *Haemophilus influenzae* (101, 108, 109, 110, 111).

The first sialic acid transport-related protein ever studied *in vitro* (111), SiaP, has now been investigated for over 15 years, with several high-resolution structures of wild-type and mutant SiaP orthologues available (Table 1), in apo- and substrate-bound forms, bound with natural sialic acids (Neu5Ac, Neu5Gc, Kdn), Neu5Ac derivatives (2,3-dehydro-2-deoxy-N-acetylneuraminic acid, aka Neu5Ac2en, sialyl-amide), or synthetic peptides, disclosing the structural basis for substrate binding (107, 108, 109, 112, 113, 114). A range of techniques including intrinsic protein fluorescence, mass spectrometry, ITC, dynamic light scattering, fluorescence resonance energy transfer, and pulsed electron–electron double resonance spectroscopies revealed the behavior and binding properties of purified SiaP in solution (105, 107, 111, 112, 113, 114). As typical of transport-associated SBPs (115), SiaP is characterized by two αβ-domains (“lobes”) connected by a hinge helix with a substrate-binding cleft located in between, which captures its substrate by bringing the lobes together through a kinking of the hinge (the so-called Venus flytrap mechanism (109, 114)). SiaP binds to Neu5Ac and Neu5Gc with high affinity with Kd figures ranging from ca 20 to 1000 nM (depending on the orthologue studied and the technique used for measurement), while Kd of 20 to 40 μM was measured for Kdn and Neu5Ac2en (105, 108, 110, 111, 114). The binding follows a bimolecular event (1:1 SiaP:Neu5AC) mediated by an induced-fit mechanism (105, 107, 111). Two conserved Arg residues (R147 and R127 in mature *Hi*SiaP from *H. influenzae* strains Rd2 and NTHi), aided by an Asn residue, coordinate the carboxylate of Neu5Ac (bound as the β-anomer) through salt bridges, and these residues are both essential for high-affinity binding (109, 113, 114), with R147 being described as a “selectivity filter” for carboxyl-containing substrates (113). Other conserved residues contribute by forming electrostatic and stacking interactions with other regions of the sugar or by stabilizing substrate-interacting residues (105, 108, 109, 114). Ordered water molecules coordinated by a single conserved residue within the binding site are also essential for high-affinity binding of Neu5Ac (112).

Once bound to Neu5Ac, SiaP delivers the substrate to the transmembrane complex SiaQM, which then transports it inside the cell (110). SiaQM forms a tight 1:1 complex in the membrane without further oligomerization (106) with transport being electrogenic and strictly dependent on both SiaP and Na^+^ based on proteoliposome studies (106, 110). The recent cryo-EM structure of *Hi*SiaQM from *H. influenzae* Rd2 (101) revealed that this complex (naturally fused in *H. influenzae*) is a structural homologue of certain elevator transporters, with a subdomain of SiaM forming the elevator domain and containing candidate substrate- and Na^+^-binding sites, and with SiaQ and the rest of SiaM dimerizing to form the so-called stator domain, which anchors the former to the membrane (101). Total internal reflection fluorescence microscopy and surface plasmon resonance studies using immobilized solubilized complexes, together with AlphaFold predictions of the tripartite PQM complex, were used to generate a model for SiaPQM’s transport cycle, where SiaP is prised open by the elevator domain, which then acquires the substrate and ferries it across the membrane in a Na^+^-dependent manner (101). This model, which was supported by the generation and characterization of bacterial mutants (101), is also confirmed in the recent cryo-EM structure of the unfused SiaQM complex from the Vibrionacea *Photobacterium profundum* SS9 (104).

##### SSS transporters

The SSS/ST5 transporter SiaT is a canonical secondary transporter comprising a single TM component, which binds its substrate directly (102, 116). A role in niche colonization for a SiaT transporter has been established for the gut pathogen *C. difficile* (SiaT was referred to as “NanT” then) (71, 117), but structural–functional information comes from the study of the orthologue from the uropathogen *Proteus mirabilis* HI4320 (*Pm*SiaT) (102). The crystal structure of *Pm*SiaT with Neu5Ac bound in an outward-open conformation (Table 1) identified a specific substrate-binding site and two Na^+^-binding sites of which one is novel and unique to SiaT-like proteins (102). The 13 TMH transporter adopts the well-known LeuT-fold with two inverted repeats of five TMH forming its core (102). Within the substrate-binding site near the center of the protein, the carboxylate of β-Neu5Ac is salt-bridged by a conserved Arg residue and H-bonded by a conserved Thr-Ser pair, which are essential interactions as demonstrated by proteoliposome-based [^3^H]-Neu5Ac uptake assays of *Pm*SiaT Ala mutants (102). Other residues confirmed by mutagenesis are essential for interacting with the substrate and/or for Na^+^ coordination (102). ITC and microscale thermophoresis determined K_D_ for the binding of *Pm*SiaT to Neu5Ac, Neu5Gc, and Kdn to be ca 50 to 60 μM, 85 μM, and > 10 mM, respectively. Transport of Neu5Ac by *Pm*SiaT is electrogenic with a proposed 2:1 ratio for Na^+^:Neu5Ac (102). That of *Pm*SiaT is the only structure solved so far, but the SiaT orthologue from the opportunistic pathogen *Staphylococcus aureus* RF122 (*Sa*SiaT) has also been studied *in vitro* (116), showing that it functions as a monomer. A recent study of proteoliposome-reconstituted *Pm*- and *Sa*SiaT transporters reported the development of Neu5Ac-based competitive inhibitors of sialic acid uptake (118), which is the first report of this kind for any prokaryotic transporter.

##### ABC transporters

After SiaPQM and SiaT, the best-characterized sialic acid transporters belong to the ABC superfamily. These are multicomponent systems that include an SBP, a dimeric TM domain, and two cytoplasmic ATPase domains (also known as NBDs for nucleotide-binding domains) that energize transfer (Fig. 4). Of three phylogenetically distinct families of ABC sialic acid transporters (SAT/ST3/SatABCD, SAT2/ST7/SatXYZ, and SAT3/ST6/SatEFG (97)), biochemical and structural information is limited to the SBP component of SAT/ST3 and SAT2/ST7 transporters.

SatA is the SBP of the SatABCD transporter found in the gut commensal bacteria *B. breve* UCC2003 (with genetic evidence for function) and *B. longum* sbps*. infantis* (97). However, to date only the orthologue from the pathogenic Pasteurellacea *Haemophilus ducreyi* 35000HP (the causative agent of the sexually transmitted disease cancroid), *Hd*SatA, has been studied at the protein level (119) (Table 1). Crystal structures are available for apo-, Neu5Ac-, and Neu5Gc-bound *Hd*SatA (119). Like SiaP (see above), SatA displays the bilobed structure characteristic of SBP (119), although sequence and structural homology is closer to peptide/oligopeptide-SBPs rather than SiaP (97, 119). *Hd*SatA uses no salt bridges to coordinate the substrate, with H-bonds and hydrophobic interactions accounting for the entire network (no differences seen between Neu5Ac- and Neu5Gc-bound forms) (119). Two H-bonds with a conserved His-Ser pair (essential for high-affinity binding) make contact with β-Neu5Ac carboxylate (119). The single conserved Arg within the binding site, also crucial for function, forms an H-bond with the glycerol tail of Neu5Ac (119). *Hd*SatA binds to Neu5Ac and Neu5Gc with Kd values of 133 and 277 nM, respectively, as determined by ITC (119) which is comparable with those of SiaP orthologues (108, 111).

The other ABC transporter functionally characterized to date is the SAT2 system discovered in some strains of the gut symbiont *R. gnavus*. This is the first transporter of any superfamily being discovered with a unique specificity for 2,7-anhydro-Neu5Ac produced by *R. gnavus* ATCC 29149 IT-sialidase (see section Sialidases) (31). Using intrinsic protein fluorescence, ITC, and STD NMR, SAT2-*Rg*SBP (also named SatX in other species; (97)) was shown to bind 2,7-anhydro-Neu5Ac with a Kd of 1.3 to 2.4 μM while no interaction was detected with Neu5Ac. Although no crystal structure is yet available, differential epitope mapping-STD NMR experiments allowed definition of the orientation of 2,7-anhydro-Neu5Ac within the binding site (31).

No biochemical/structural information is available for the third family of ABC sialic acid transporters, *i.e.*, the SAT3/ST6/SatEFG family, which is genetically characterized in *Streptococcal* species, and is also found in the gut commensal bacteria *Ruthenibacterium lactatiformans* and *Roseburia inulinivorans* (97, 120).

##### MFS transporters

There are three phylogenetically distinct families of MFS sialic acid transporters, namely, ST1/NanT/NanX (found in *E. coli* and *Salmonella typhimurium* strains), ST4/NanZ (found in *B. fragilis* and several other gut *Bacteroidetes*), and ST8/NanG (found in *Ligilactobacillus salivarius* LMG14477) (Fig. 4, (97)). While NanG is not experimentally characterized, genetic studies have shown that the others function as proton-dependent sialic acid transporters (97), which are specific for either Neu5Ac (NanT and NanZ; (97)) or 2,7-anhydro-Neu5Ac/Neu5Ac2en (NanX; (100, 121)). However, there are no biochemical studies for any of these transporters, except for a SiaPQM-focused work that compared SiaPQM- with NanT-loaded proteoliposomes for features such as ion dependence and transport reversibility (106).

#### OM sialic acid transporters

So far transport through the OM of gram-negative bacteria has received little attention and only two sialic acid uptake systems have been identified to date.

The sialoporin NanC, encoded by the first gene of the *nanCMS* operon in *E. coli*, is a monomeric 12-stranded β-barrel protein belonging to the KdgM family of sugar transport–associated porins (122) (Table 1). In proteoliposomes, Neu5Ac transport through NanC is nonsaturable and is voltage- rather than pH-gated (122, 123). The 3D structure of NanC from the *E. coli* K12 strain has been solved at high resolution (122) but no complex with ligands is available (122). *E. coli* growth assays showed that NanC can transport monomeric Neu5Ac (123); however, it is not yet clear whether sialic acid oligomers and/or sialoglycans might be the natural substrates of NanC (122, 123).

NanU is a SusD-family protein that serves as the substrate-binding protein of the Neu5Ac-specific SusCD system, NanOU, of *B. fragilis* NCTC 9343, which also includes the TonB-dependent receptor porin NanO (124). Both *B. fragilis* NanU, *Bf*NanU, and the orthologue from the oral pathogen *Tannerella forsythia* ATCC 43037 bind to Neu5Ac with a Kd of ca 400 nM, and with ca 4- to 5-fold higher values to Neu5Gc, as shown by ITC (124). NanU is specific for monomeric Neu5Ac rather than for sialoglycans (124) making it unique among the oligosaccharide-binding SusD proteins (125). The crystal structure of apo-*Bf*NanU (Table 1) shows a monomer (confirmed by gel filtration chromatography) with the typical fold of SusD proteins containing four tetratricopeptide repeats connected by loops (124). No complex structure with bound Neu5Ac is available as yet (124). The *nanOU* operon can complement for *nanC* when heterologously expressed in *E. coli* (124), confirming a role in OM Neu5Ac transport for NanOU. However, there is no structural or functional information on the OM component NanO.

Although no other OM uptake systems have been studied so far, the pervasive presence of uncharacterized *susCD* genes among sialocatabolic clusters from *Bacteroidetes* (97) suggests the existence of yet undiscovered transport systems for different forms of sialic acid and/or sialoglycans.

### Sialic acid metabolic enzymes

Once transported into the bacterial cells, sialic acid is converted into sugars *via* a set of metabolic enzymes encoded by the nan clusters, and which may differ depending on the type of sialic acids and sialic acid metabolic pathways, as described below.

#### Sialic acid metabolic pathways in gut bacteria

Two distinct metabolic pathways for Neu5Ac utilization by bacteria have been demonstrated in *E. coli* and *B. fragilis* (126), respectively, and these confer an advantage in gut colonization (127).

**Figure 5:**
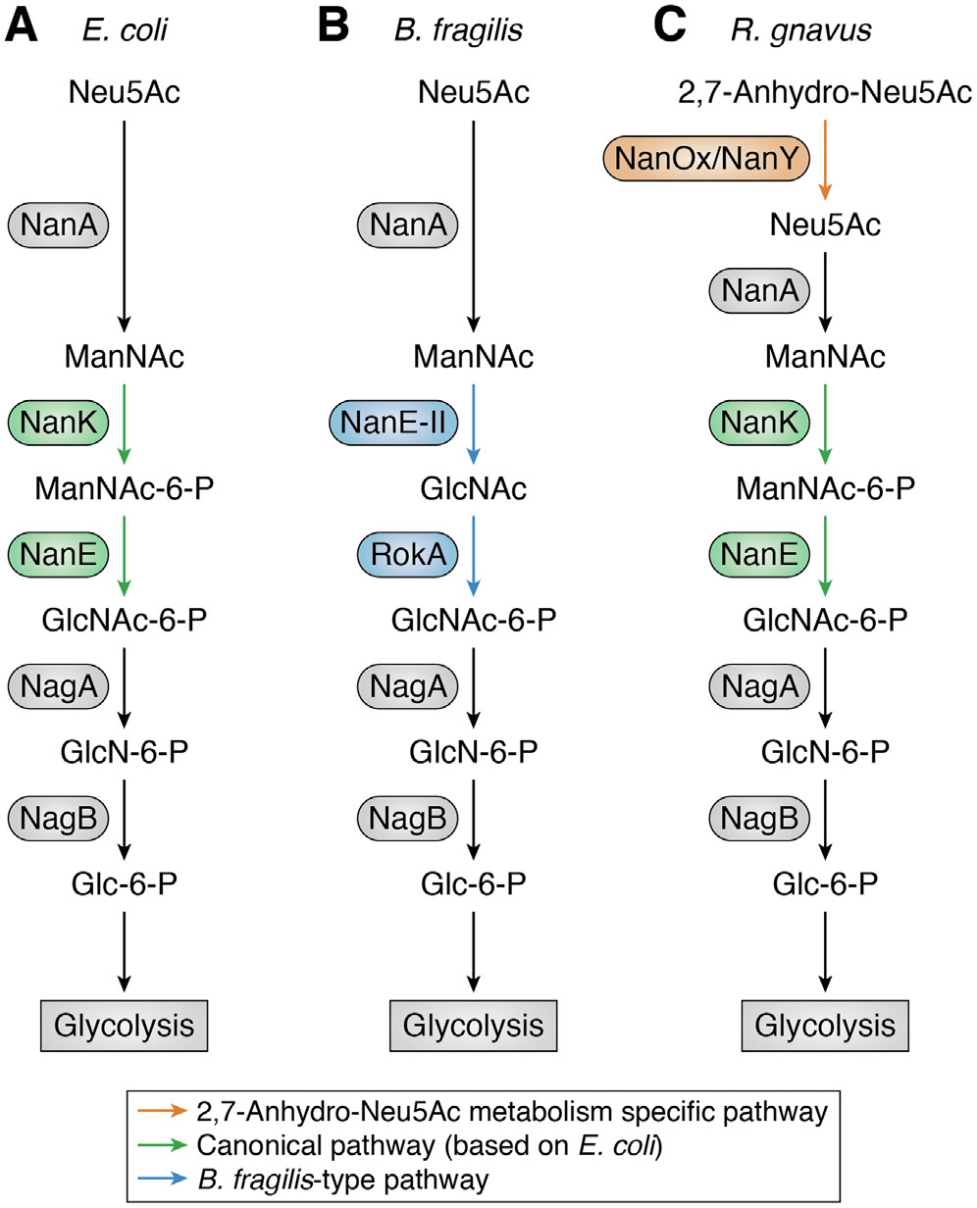
**Schematic representation of the sialic acid metabolic pathways identified in gut bacteria.***A*, *E. coli* Neu5Ac metabolism. *B*, *B. fragilis* Neu5Ac metabolism. *C*, *R. gnavus* 2,7-anhydro-Neu5Ac metabolism. Difference in the pathways are highlighted: *orange*, 2,7-anhydro-Neu5Ac metabolic pathway; *green*, “*E. coli*” Neu5Ac metabolic pathway; *blue*, *B. fragilis* Neu5Ac metabolic pathway.

The most studied pathway of utilizing sialic acid is the *nanA/K/E* cluster first described in *E. coli* (128) (Fig. 5*A*). Following transport of sialic acid residues into the cells, a sialic aldolase or lyase (NanA) cleaves sialic acid into pyruvate and N-acetylmannosamine (ManNAc), which is then phosphorylated by a ManNAc kinase (NanK) belonging to the ROK superfamily (128). The phosphorylated ManNAc is then converted to a phosphorylated GlcNAc (GlcNAc-6-P) by an epimerase (NanE). It is then successively deacetylated and deaminated by NagA (N-acetylglucosamine-6-phosphate deacetylase) and NagB (N-acetylglucosamine-6-phosphate deaminase), respectively, two enzymes not encoded within the *nan* operon but elsewhere in the genome (97). This results in fructose-6-phosphate, which then enters the glycolysis pathway, while the by-products from the enzyme reactions can be used as precursors in a number of important cellular processes (129). Similar *nanA/K/E* metabolic clusters with differences in transporter mechanism have been identified in a range of bacteria, including commensal bacteria (*Bacteroides*, *Bifidobacterium*, *Blautia*, *Clostridium*, *Faecalibacterium*, *Flavonifractor*, *Fusobacterium*, *Parabacteroides*, and *Prevotella* genera) and pathogenic species (*Clostridioides*, *Staphylococcus*, and *Streptococcus*) (37).

An alternative pathway has later been discovered in *B. fragilis* (Fig. 5*B*). Here following transport of sialic acid into the cell, a sialic acid lyase, NanL, which is from a distinct NanA clade (130), cleaves Neu5Ac into pyruvate and ManNAc. Then, NanE-II, which is dissimilar to the NanE found in the *nanA/K/E* cluster, directly epimerizes ManNAc to GlcNAc without the need for prior phosphorylation. An alternative ManNAc kinase (RokA) then performs the phosphorylation before NagA and NagB, again, converts it to fructose-6-phosphate (126). This pathway can also be identified in a range of gut microbes particularly from the *Bacteroidetes* phylum (68).

These sialic acid metabolic pathways have mainly been identified and studied in the context of Neu5Ac metabolism; however, recent transcriptomics and biochemical studies identified a sialic acid metabolic pathway specific to 2,7-anhydro-Neu5Ac in *R. gnavus* strains (31, 63, 100, 131) (Fig. 5*C*). Both ATCC 29149 and ATCC 35913 strains *R. gnavus* strains were able to grow on 2,7-anyhydro-Neu5Ac, the IT-sialidase transglycosylation product, as a sole carbon source (131). Following 2,7-anhydro-Neu5Ac transport into the cell *via* a specific transporter, 2,7-anhydro-Neu5Ac is converted back to Neu5Ac *via* an oxidoreductase, *Rg*NanOx, which then enters the canonical NanA/K/E pathway described above.

The following section provides an overview of the biochemical and structural properties of the main sialic acid metabolic enzymes encoded by the Neu5Ac and 2,7-anhydro-Neu5Ac metabolic pathways described above.

#### Sialic acid oxidoreductases

**Figure 6:**
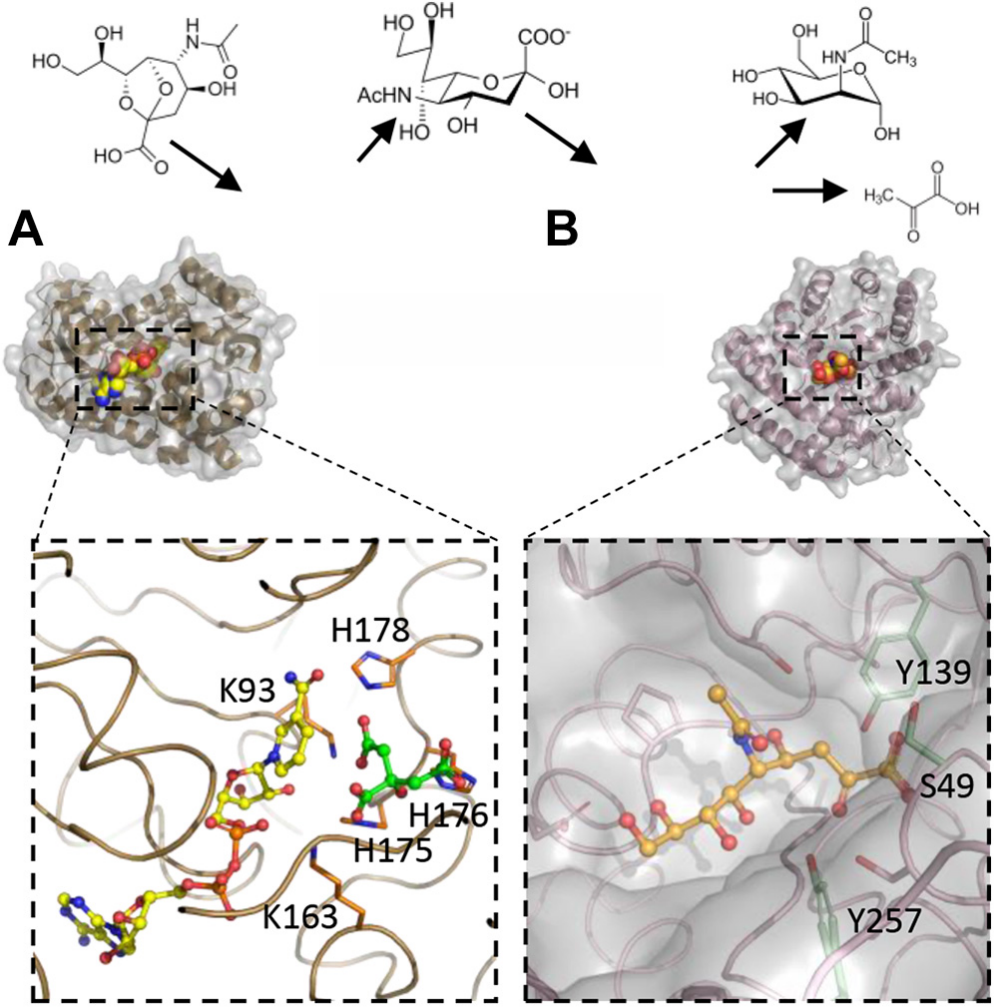
**Structural characteristics of 2,7-anhydro-Neu-5Ac metabolic enzymes.** Once transported inside *R. gnavus* ATCC 29149 cells (*A*) *Rg*NanOx converts 2,7-anhydro-Neu5Ac into Neu5Ac with the aid of an NAD cofactor. The NAD cofactor and a bound citrate molecule are shown in *yellow* and *green*, respectively (PDB 6Z3C). *B*, Neu5Ac is subsequently converted to ManNAc and pyruvate by *Rg*NanA. Neu5Ac in its open-chain ketone form is shown bound in the active site with catalytic residues in green (PDB 6RD1). For clarity, the surface representation has been omitted from the active site due to its buried nature. PDB, Protein Data Bank.

As mentioned above, for 2,7-anhydro-Neu5Ac to be metabolized into the cells, it must first be converted by *Rg*NanOx (Table 1) into Neu5Ac through a 4-keto-2-deoxy-2,3-dehydro-N-acetylneuraminic acid intermediate, as demonstrated in *R. gnavus* ATCC 29149 (100). The crystal structure of *Rg*NanOx showed a typical Rossman fold, characterized by a central β-sheet with helices on either side (Fig. 6*A*). Once 2,7-anhydro-Neu5Ac is converted into Neu5Ac, this becomes a substrate for *Rg*NanA, which catalyzes the release of pyruvate and ManNAc. It was also discovered that some strains of *E. coli* possess a homologue of *Rg*NanOx, YjhC (NanY), which can also catabolize this reaction and is essential for *E. coli* to metabolize 2,7-anhydro-Neu5Ac (Table 1) (100). Predicted homologues of this oxidoreductase were identified in a range of microbial species, suggesting a diversity in 2,7-anhydro-Neu5Ac utilization (97). Owing to similarities with the intermediate structure of the NanOx reaction mechanism, NanOx could also play a role in the metabolism of Neu5Ac2en (100), although this remains to be tested experimentally beyond *E. coli* (132).

#### Sialic acid aldolases

Sialic acid aldolases are the first metabolic enzymes in sialic acid metabolic pathways. Sialic acid aldolases are well conserved across multiple species, and crystal structures from a range of species show a conserved (β/α)_8_ TIM barrel with an adjacent three-helix bundle (31, 133). To date sialic acid aldolases have been functionally characterized from species including *Pasteurella multocida*, *Clostridium perfringens*, *Lactobacillus plantarum*, *E. coli*, and *R. gnavus* ATCC 29149 (134, 135, 136, 137, 138) (Table 1). The sialic acid aldolase reaction mechanism is reversible and proceeds through formation of a Neu5Ac–Schiff base with an active-site lysine residue. This mechanism requires Neu5Ac to be in the ring open form. Recently, it was discovered that an anomerase, YhcH, (NanQ) from *E. coli* K12, can catalyze the ring opening providing a linear substrate for sialic acid aldolases (Table 1) (132). Homologues of YhcH are often found in sialic acid metabolism from various organisms of different phyla (97), suggesting an important evolutionary adaptation.

The substrate specificity of sialic acid aldolases has mainly been investigated toward Neu5Ac. However, a sialic acid aldolase from *Sphingobacterium* sp. has recently been shown to be active toward Kdn but not Neu5Ac or Neu5Gc (139). Site-directed mutagenesis of the Asp50 residue in *Sphingobacterium* sp. NanA showed that it was important for Kdn specificity. However, to date there are no reports of sialic acid aldolase using other Neu5Ac derivatives produced by gut microbes such as 2,7-anhydro-Neu5Ac (32). Indeed it was shown that, although *R. gnavus* ATCC 29149 can transport 2,7-anhydro-Neu5Ac into the cells due to exquisite substrate specificity of the sialic acid transporter, the sialic aldolase, *Rg*NanA, is specific for Neu5Ac and does not utilize 2,7-anhydro-Neu5Ac as a substrate, as also confirmed by the crystal structure of the complex showing Neu5Ac in the open-chain ketone form, with the N-acetyl group oriented out of the active site (31) (Fig. 6*B*).

#### Sialic acid kinases and epimerases

Following the conversion of Neu5Ac into ManNAc, NanK, phosphorylates ManNAc into ManNAc-6-P (see Fig. 5). The crystal structure was solved for the NanK enzymes from *F. nucleatum* and *S. aureus* strains, which showed that, in contrast to other ROK family kinases, the conserved zinc-binding site is absent in these NanK orthologues (140, 141) (Table 1). Expanding on these results, a later study (142) comparing *in vitro* catalysis by these two zinc-independent NanK proteins with that of the zinc-binding orthologues from *H. influenzae*, *P. multocida*, and *V. cholerae* strains, found that, although some enzymatic properties may vary among orthologues (such as K_M_, k_*cat*_, or the order by which ManNAc and ATP enter the binding site), these did not correlate with the presence or absence of the zinc-binding site. Additional structures of *Pm*NanK from *P. multocida* and *Hi*NanK from *H. influenzae*, with various substrates or products, provided more insights into the catalytic mechanism of these enzymes (142). Following phosphorylation, the epimerase enzyme (NanE) performs the conversion to GlcNAc-6P. A one-base catalytic mechanism involving the deprotonation and reprotonation of C2 *via* an enolate intermediate structure mediated by a catalytic lysine was described based on the crystal structure of NanE from *C. perfringens* strain 13 (143) (Table 1). The alternative pathway of ManNAc metabolism found in *Bacteroidetes* proceeds through the NanE-II enzyme (Table 1), which has been shown to be an AGE family epimerase *in vitro* (126). This produces GlcNAc, which is then phosphorylated by the RokA kinase, which has been shown to have a broad substrate specificity (Table 1) and is indeed involved in the metabolic pathways for other N-acetyl-sugars (144). These are then further processed by NagA and NagB, which are generally recruited from the GlcNAc/ManNAc pathway resulting in fructose 6-phosphate, which then enters the glycolysis pathway (see Fig. 5).

## Concluding remarks

The functional studies outlined above collectively indicate that gut bacteria have evolved multiple pathways for releasing, transporting, and metabolizing sialic acid derivatives, underscoring the importance of sialic acid as a precious nutrient in the adaptation of microbial communities to the gut. Although most studies to date focused on Neu5Ac as the main form recognized by sialidases, sialic acid transporters, or sialic acid aldolases, studies are emerging showing specificities to other forms of sialic acid produced by gut bacteria such as 2,7-anhydro-Neu5Ac. In addition, most biochemical and structural knowledge on sialic transporters to date is derived from studies of pathogens or microbes from other ecological niches. Given the wide structural diversity of sialic acid forms in the gut, it is likely that many more pathways will be unraveled in the years to come. Alterations in sialic acid homeostatic levels in the gut have been associated with infection and inflammation in preclinical models, but underpinning mechanisms remain to be uncovered. With the field of gut microbiota expanding beyond association studies, and the acknowledgement of sialic acid as a key mediator of human health, it is critical to expand our mechanistic insights into the range of sialic acid derivatives metabolized by gut microbes, and their role in signaling within and outside the gut. This will no doubt continue to advance our understanding of the coevolution of humans with their microbes while providing novel biomarkers and therapeutic targets.

## Conflicts of interest

The authors declare that they have no conflicts of interest with the contents of this article.
